# Red blood cell transfusion in patients with traumatic brain injury: a systematic review

**DOI:** 10.1186/cc14412

**Published:** 2015-03-16

**Authors:** A Boutin, M Chasse, M Shemilt, F Lauzier, L Moore, R Zarychanski, J Lacroix, DA Fergusson, D Griesdale, P Desjardins, AF Turgeon

**Affiliations:** 1 Universite Laval, Quebec, QC, Canada; 2 University of Manitoba, Winnipeg, MB, Canada; 3 Universite de Montreal, QC, Canada; 4 Ottawa Hospital Research Institute, Ottawa, ON, Canada; 5 University of British Columbia, Vancouver, BC, Canada

## Introduction

We aimed to evaluate the frequency of red blood cell (RBC) transfusion in patients with traumatic brain injury (TBI) as well as determinants and outcomes associated with RBC transfusion in this population.

## Methods

We conducted a systematic review of cohort studies and trials of patients with TBI. We searched Medline, Embase, The Cochrane Library and BIOSIS databases from their inception up to 30 June 2014. We selected cohort studies and RCTs of adult patients with TBI reporting data on RBC transfusions. We extracted data related to demographics, baseline characteristics, blood product use and any relevant clinical patient-oriented outcome. Cumulative incidences of transfusion were pooled through random effect models with a DerSimonian approach, after a Freeman-Tukey transformation to stabilize variances. To evaluate the association between RBC transfusion and potential determinants as well as outcomes, we pooled risk ratios or mean differences with random effect models and the Mantel-Haenszel method. Sensitivity and subgroup analysis were planned *a priori*.

## Results

We identified 21 eligible studies (16,951 patients). After pooling data from the 20 included cohort studies (16,884 patients), at least around 33% (95% CI: 27 to 39; *I*^2^: 97.8%) of patients with TBI in published reports received transfusions at some point during their hospital stay. In a *post hoc *analysis of one RCT comparing transfusion strategies, 82% of patients were transfused RBCs. Thresholds for transfusion were rarely available and varied from 6 to 10 g/dl. From raw data, Glasgow Coma Scale scores were lower in patients who were transfused than those who were not (three cohort studies; n = 1,371; mean difference of 1.38 points (95% CI: 0.86 to 1.89); *I*^2 ^= 12%). Mortality was not significantly different among transfused and nontransfused patients both in univariate and multivariate analyses. Hospital length of stay was longer among patients who were transfused (three studies; *n *= 455; mean difference 9.58 days (95% CI: 3.94 to 15.22); *I*^2 ^= 74%). Due to the observational nature of included studies, results should be considered cautiously due to the high risk of confounding.

## Conclusion

RBC transfusion is frequent in patients with TBI, but practices varied widely in cohort studies in this population. The paucity of data precludes definitive conclusions and highlights the lack of clinical evidence guiding transfusion strategies in TBI.

